# Assessing the Efficacy of the Modified SEGA Frailty (mSEGA) Screening Tool in Predicting 12-Month Morbidity and Mortality among Elderly Emergency Department Visitors

**DOI:** 10.3390/jcm12226972

**Published:** 2023-11-07

**Authors:** Abrar-Ahmad Zulfiqar, Mathieu Fresne, Emmanuel Andres

**Affiliations:** 1 Geriatric Readaptation Unit, Vitry-Le-François Hospital, 51300 Vitry-Le-François, France; 2 General Medicine Department, University Hospital of Reims, 51100 Reims, France; mathieufresne@gmail.com; 3 Internal Medicine Department, University Hospital of Strasbourg, 67000 Strasbourg, France; emmanuel.andres@chru-strasbourg.fr

**Keywords:** mSEGA frailty scale, emergencies, elderly subjects, prognostic factor

## Abstract

Introduction: Rapid identification of frail elderly individuals upon admission to the emergency department is pivotal for enhancing their care and alleviating emergency room congestion. Objective: This pilot study aims to explore the relationship between morbidity, mortality, and frailty, as assessed by the mSEGA scale, among individuals aged 65 years or older in the emergency department. Methods: A retrospective cohort study was conducted at a single center. The pilot study included patients aged 65 and above who were admitted to Chaumont Hospital’s emergency unit (Haute-Marne department) for medical and/or surgical reasons between 1 July 2017 and 31 January 2018. Data encompassed socio-demographic characteristics, medical profiles, and emergency department visit details. Outcomes for patients one year post-admission were obtained through consultation with their respective general practitioners. Results: A total of 255 subjects participated, with a mean age of 82.1 ± 8.2 years. Primary admission reasons were falls (*n* = 51, 20.0%), digestive issues (excluding hemorrhage) (*n* = 30, 11.8%), and “other” causes (*n* = 61, 23.9%). Among participants, 78 (30.6%) scored ≤8 on the mSEGA frailty scale, 49 (19.2%) scored 9 to 11, and 125 (50.2%) scored ≥12. Concerning post-emergency department outcomes, 152 patients (59.6%) were hospitalized, while 103 (40.4%) were discharged. No deaths were reported during the study period, and vital status was known for all subjects at the one-year mark. At that point, 63 out of 255 patients had passed away, with 30 of them being readmitted to the emergency department either before or at the time of their one-year death. The 12-month survival rate analysis based on frailty status revealed a significant difference. Low-frailty patients exhibited a survival rate of 87.2% (95% CI; [77.5–92.9]), whereas frail/very frail patients had a survival rate of 70.0% (95% CI; [62.7–76.2]). Similarly, the 12-month readmission-free survival rate demonstrated statistically significant disparities. Low-frailty patients had a rate of 76.9% (95% CI; [65.9–84.8]), compared to 51.4% (95% CI; [43.8–58.5]) for very frail patients. Conclusion: Utilizing the mSEGA frailty scale in the Emergency Department could provide crucial prognostic insights, highlighting significant differences in 12-month survival and readmission-free survival rates based on frailty status.

## 1. Introduction

In France, the population of individuals aged 75 and above currently stands at 5.6 million people, and this number is expected to rise in the coming years. According to demographic projections by the French National Institute of Economic and Statistical Information (INSEE), there is estimated to be a 50% increase in the number of potentially dependent elderly individuals between 2000 and 2040, reaching a total of 10 million people [1].

Among geriatric patients, admission to the emergency department is the most common method of hospital admission. Currently, geriatric patients account for 15 to 20% of the total admissions to emergency departments. With ongoing demographic changes, this number and proportion of geriatric admissions are anticipated to further increase [2].

In 2011, the French Society of Gerontology and Geriatrics defined frailty syndrome as a clinical syndrome characterized by a reduction in physiological reserves across multiple systems, rendering an elderly person susceptible to adverse effects when subjected to stress. The clinical manifestation of frailty is influenced by comorbidities as well as psychological, social, economic, and behavioral factors [3]. In 2003, the jury of the 10th Consensus Conference of the French Society of Emergency Medicine (SFMU) defined frailty in elderly persons as the “risk of imbalance between somatic, psychic, and social elements, provoked by even minimal aggression [4]. In practice, frailty is manifested and evaluated by the appearance of cognitive, behavioral, and sensory disorders, polypathologies, polypharmacy, and by an increased need for assistance in daily living. Frailty can be patent or latent”. The concept of frailty is complex because individuals react differently to the stressors they encounter. Although there is no universally agreed-upon definition of frailty, it is widely accepted that frailty exposes elderly patients to unfavorable health outcomes, including unplanned hospitalizations, institutionalization, increased morbidity, and mortality.

In a three-year follow-up study, Fried et al. found that frail elderly individuals had a six-fold higher risk of mortality, a five-fold higher risk of dependency, and approximately a two-fold higher risk of falling compared to non-frail elderly individuals [5]. Similarly, Rockwood et al. reported a nine-fold increased risk of institutionalization among frail individuals [6]. The SAFEs study also revealed that pre-frail and frail patients experienced longer hospital stays and higher rates of re-hospitalization [7,8]. Considering that the proportion of the French population aged 75 years and older is continuously rising and projected to double between 2010 and 2060 [9], it is crucial to identify and detect frailty in order to prevent these complications and reduce associated expenses.

Emergency physicians play a crucial role in providing hospital care for elderly patients, regardless of the reason for their admission. In the scientific literature, various scoring systems have been developed to assist emergency physicians in identifying frailty in elderly individuals. One such scale is the modified SEGA score (Short Emergency Geriatric Assessment), which was developed by a team of Belgian researchers [10]. The mSEGA frailty scale is a multidimensional scale that is based on the concept of frailty as defined by the work of Rockwood [6].

Thus, the main objective of this study was to study the relationship between frailty, as evaluated by the mSEGA frailty scale in the emergency department, and morbi-mortality.

## 2. Methods

### 2.1. Study Design and Population

A monocentric retrospective cohort study was conducted. The study included patients aged 65 years and older, admitted to the emergency department of the Chaumont Hospital Center (Haute-Marne department, France) for medical and/or surgical reasons between 1 July 2017, and 31 January 2018, and who did not object to participating. The exclusion criteria comprised of patients receiving invasive medical techniques, those with a life-threatening prognosis, patients under palliative or end-of-life care, individuals objecting to participation in the study, those protected by law (under guardianship, curatorship, or safeguard of justice), and cases where it was impossible to retrieve the mSEGA frailty score from the medical records of Chaumont Hospital’s emergency department.

### 2.2. Data Collected

The study collected various data for each patient, including sociodemographic characteristics such as age, sex, and living arrangements (whether they resided in a personal residence or nursing home) and the presence of formal assistance. Medical data were also collected, including the patients’ medical and surgical history, current treatments, and the presence of polypharmacy, defined as the intake of six or more medications. The Charlson Comorbidity Index score, which assesses the burden of comorbidities, was also recorded for each patient. Data regarding the visit to the emergency department were also gathered, including the reason for referral and the method of referral. The method of referral could be either the patient themselves, the attending physician, the Dial 15 emergency call center dispatch, or other means. Lastly, the outcome after the visit to the emergency department was recorded, including whether the patient was discharged to home, hospitalized, or deceased.

The frailty status of the patient was evaluated using the Short Emergency Geriatric Assessment (SEGA) from the patients’ medical records. For the mSEGA frailty score, the patients were assigned to two groups: non-frail patients (score < 8), and frail or very frail patients (score > 8). The mSEGA frailty scale assesses frailty using a 13-item scale that includes the following components: medications, mood, perception of health, fall in the previous 6 months, nutrition, associated diseases, mobility, continence, cognitive function, age, place of living, instrumental activities of daily living (IADL), and meals. Each item is assigned a score of 0 (indicating the most favorable state) or 1 or 2 (indicating the least favorable state). The scores from each item are summed, resulting in a total score ranging from 0 to 26. Based on the total score, individuals can be categorized into three groups: not very frail (score ≤ 8), frail (score > 8 and ≤11), and very frail (score > 11).

We decided to merge the categories of very frail and frail patients because it seemed crucial to distinguish robust patients without pathological gerontological criteria from frail patients who presented with one or more abnormal geriatric criteria. Although there is limited scientific literature on the modified SEGA scale, previous studies utilizing this scale have analyzed frailty by grouping patients into two categories: non-frail and frail/very frail. According to the mSEGA frailty scale, the non-frail or robust patient had a score between 0 and 8/26, and frail and very frail patients had a score above or equal to 9/26.

The frailty assessment in this study was performed by a team of experienced healthcare professionals, including physicians and nurses, who were trained in the use of the modified Short Emergency Geriatric Assessment (mSEGA) scale. These healthcare providers were responsible for conducting the assessments as part of the routine clinical evaluation of older adults upon admission to the emergency department. The mSEGA scale, a validated tool for frailty assessment, was integrated into the standard assessment protocol to ensure consistency and accuracy in frailty identification.

By involving healthcare professionals familiar with the emergency department environment, we aimed to replicate real-world clinical practice and ensure the feasibility of incorporating frailty assessment into the existing workflow. This approach reflects the pragmatic nature of our study and its potential for seamless integration into routine emergency care practices.

The relatively small sample size (N = 255) over a nine-month period in the emergency unit can be attributed to the opportunistic nature of our recruitment process. Rather than conducting a planned recruitment effort, we leveraged the unique opportunity presented during the course within this clinical unit. This approach allowed us to collect data from patients as they presented to the emergency department, thereby reflecting a real-world, unselected patient population. While this opportunistic recruitment strategy offered valuable insights into the relationship between frailty and health outcomes in this setting, it may have limitations in terms of sample size compared to more structured recruitment methods.

The outcome of patients at 12 months after their admission to the emergency department was assessed by contacting their general practitioners. The events of interest were death from any cause, institutionalization, readmission to the Chaumont Hospital emergency department, and the provision of home care.

### 2.3. Ethical and Legal Aspects

Consent was collected for all individuals included in the study. To collect and manage the data, a database was created using Limesurvey^®^ software (Copyright © 2006–2023 LimeSurvey GmbH- github.com/LimeSurvey) hosted by the University of Reims Champagne Ardenne. The recorded data were pseudonymous. Data processing complied with current French regulations, including the General Data Protection Regulation 2016/679 of the European Parliament and the Council of 27 April 2016, which has been in effect since 25 May 2018 (GDPR), as well as the French Data Protection Act of 6 January 1978, amended in 2018. The study was also registered on the Health Data Hub platform (no. F20220316120856).

### 2.4. Statistics

Continuous variables were expressed as mean ± standard deviation or using the median and interquartile range. Categorical variables were expressed as numbers and percentages.

Comparisons between patient characteristics and the evaluation criteria (institutionalization within 12 months, initiation of home care within 12 months, and readmission within 12 months) were performed using the Chi^2^ test (or Fisher’s exact test) for categorical variables, while the Student’s *t*-test was used for continuous variables. For each evaluation criterion, factors associated with a *p*-value < 0.10 in the univariate analysis were then entered into a top-down multivariate selection logistic regression model with a cutoff set at 0.05.

Survival time was defined as the time from the date of arrival in the emergency department to the date of death. Patients who were still alive at the end of follow-up were screened at their last follow-up date or at the study’s endpoint. Median survival time was compared by patient characteristics using a log-rank test. Factors associated with survival (*p* < 0.10) were then included in a multivariate Cox hazard ratio model with stepwise variable selection and a cutoff point of 0.05. The hazard ratio (HR) and 95% confidence interval were obtained from the model. The hazard ratio assumption was tested by examining the Schoenfeld residuals.

Survival curves were constructed using the Kaplan–Meier method. For assessing the normality of the distribution, the Shapiro–Wilk test was used.

All statistical tests were performed at the 0.05 level of significance. The data were analyzed using SAS version 9.4 software (SAS Institute, Cary, NC, USA).

## 3. Results

### 3.1. Characteristics of the Study Population

A total of 255 patients were included in the study. The characteristics of the population are described in Table 1. The mean age of the patients was 82.1 ± 8.2 years, and 56.9% were women. Among them, 52.8% were admitted to the emergency department following a call to the emergency call center, 24.0% came on their own without prior medical advice, and 21.3% of patients were referred to the emergency department by their attending physician. The most frequent reasons for admission were falls (20.0%), digestive problems (excluding digestive hemorrhages) (11.8%), and general deterioration (11.0%). The average Charlson Comorbidity Index was 5.5 ± 2.0. Polypharmacy was present in 71.9% of patients. Patients living at home represented 82.8% of the total number of patients; 35.3% had formal home care.

According to the mSEGA frailty scale, 30.6% of patients were classified as “low frailty”, 19.2% as “frail”, and 50.2% as “very frail”. Following the emergency department visit, 40.4% of patients returned home and 59.6% were hospitalized.

### 3.2. Institutionalization at 12 Months

Of the 210 patients for whom information could be collected, 11 (5.2%) were institutionalized within 12 months of their visit to the emergency department (Table 2).

In the univariate analysis (Table 3), only frailty tended to be associated with institutionalization at 12 months: 10/11 (90.9%) of institutionalized patients were frail or very frail versus 122/199 (61.3%) of non-institutionalized patients (*p* = 0.06). A multivariate analysis was not performed because only one factor tended to be associated with institutionalization in the univariate analysis.

### 3.3. Initiation of Home Care at 12 Months

Among the 120 patients for whom information could be collected, home care was initiated within 12 months of the emergency department visit for 19 (15.8%) patients.

In the univariate analysis (Table 4), no factor was associated with the initiation of home care at 12 months. Frailty was not associated with the initiation of home care at 12 months: 10/72 low-frailty patients (13.9%) received home care at 12 months versus 9/48 frail and very frail patients (18.8%) (*p* = 0.47) who received home care at 12 months.

### 3.4. Emergency Department Readmissions at 12 Months

Among the 254 patients for whom information could be collected, 71 (27.9%) had been readmitted to the emergency department within 12 months of their first emergency department visit.

In the univariate analysis (Table 5), frailty was significantly associated with readmission to the emergency department within 12 months: (59/71) 83.1% of patients who were readmitted to the emergency department within 12 months were frail or very frail versus 117/183 (63.9%) of patients who were not readmitted to the emergency department within 12 months (*p* = 0.003). The Charlson score ((5.9 ± 2.4) for patients readmitted to the emergency department within 12 months versus 5.4 ± 1.8 for patients not readmitted to the emergency department within 12 months (*p* = 0.07)) and polypharmacy ((56/71) (80, 0%) of patients readmitted to the emergency department within 12 months versus 125/183 (68.7%) of patients not readmitted to the emergency department within 12 months (*p* = 0.07)) tended to be associated with readmission to the emergency department within 12 months.

In the multivariate analysis (Table 5), only frailty was associated with readmission to the emergency department within 12 months (OR = 2.71 [1.36–5.41]; *p* = 0.005).

### 3.5. Death at 12 Months

**Figure 1:**
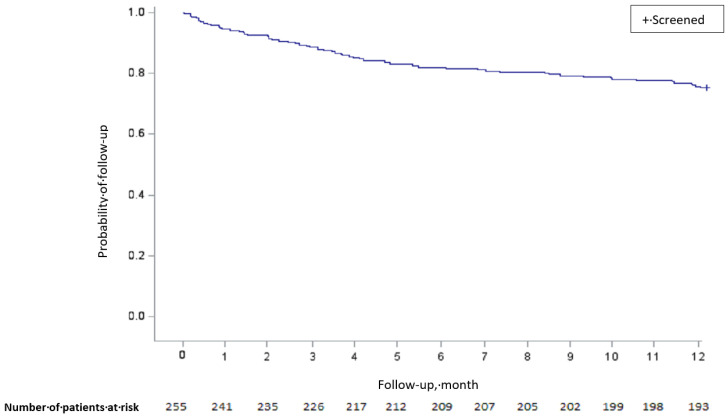
Kaplan–Meier survival curve for the entire population.

Of the 254 patients for whom information could be collected, 62 (24.4% [95% CI, 19.4–30.0]) died within 12 months (Figure 1).

**Figure 2:**
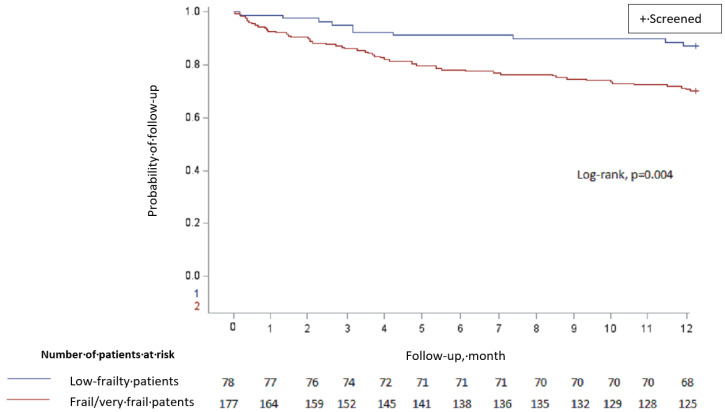
Twelve-month survival curve for low-frailty and frail/very frail patients.

In the univariate analysis (Table 6), polypharmacy (*p* = 0.03), frailty (*p* = 0.006), and the Charlson score (*p* < 0.0001) were found to be significantly associated with death within 12 months. The survival curve according to frailty status (frail or very frail versus low-frailty patients) is presented in Figure 2.

In the multivariate analysis (Table 6), only the Charlson score was significantly associated with death at 12 months (HR = 1.26 [1.14–1.39]; *p* < 0.0001).

## 4. Discussion

The primary objective of this study was to evaluate the predictive capacity of the mSEGA frailty scale in identifying elderly patients at risk of readmission to the emergency department, institutionalization, initiation of home care, and death within 12 months after their emergency department visit. Notably, this research is a pilot study which represents a novel exploration of the mSEGA frailty scale within the context of an emergency department, a setting that has received limited attention in previous research, which has predominantly focused on elderly patients in ambulatory care, home settings, and geriatric wards.

Our investigation centered on examining the relationship between frailty as assessed by the mSEGA scale and key outcomes, including morbidity and mortality. While we identified various trends in our data, we emphasize statistically significant results to provide a clearer understanding of frailty’s implications within this context.

A noteworthy finding of our study was the connection between frailty status and mortality within the 12-month period post-emergency department admission. Specifically, we observed that patients with low frailty had a significantly higher 12-month survival rate compared to frail/very frail patients, aligning with the established understanding of frailty as a risk factor for adverse health outcomes in elderly populations. It is worth noting that our study did not report any deaths during the study period, possibly due to its relatively short follow-up duration. Nevertheless, the statistically significant difference in survival rates underscores the clinical relevance of frailty assessment in the emergency department.

Another significant aspect of our findings pertains to the link between frailty status and readmission to the emergency department within 12 months. Low-frailty patients demonstrated a notably higher readmission-free survival rate compared to very frail patients, suggesting that frailty assessment may be a valuable tool in identifying individuals at greater risk of recurrent emergency department visits. A study conducted by Leblanc et al. [11] involved a prospective, single-center cohort study of patients discharged from a geriatric hospital ward. The study followed the patients for six months and demonstrated that the modified SEGA scale had predictive capabilities for adverse events such as readmission to the hospital at three months and multiple falls at six months. Tailoring interventions and care plans for very frail patients could potentially mitigate readmission risk and enhance overall care quality for this vulnerable population.

It is important to reiterate that our primary focus was on statistically significant results to ensure the robustness of our findings. Statistically significant differences in survival and readmission rates based on frailty status underscore the mSEGA scale’s potential clinical utility in risk stratification and patient management.

Our results suggest that the tool can anticipate mortality, but that should be confirmed with prospective and multicenter studies. Our pilot study contributes novelty to the fields of emergency medicine and gerontology by examining the relationship between frailty, mortality, and readmission specifically within the emergency department context. However, these results must be confirmed in a multicentric prospective series in the future.

While frailty has been extensively studied in other healthcare settings, such as long-term care or inpatient facilities, its relevance in the emergency department has received comparatively less attention. Our research fills this gap by focusing on a unique and understudied population—older adults seeking emergency care.

The findings of this study have substantial health implications for both healthcare providers and older adults in emergency care. By identifying frailty as a predictor of adverse outcomes, including mortality and readmission, we underscore the importance of incorporating frailty assessment into the initial evaluation of older patients in the emergency department. Early detection of frailty can lead to tailored interventions addressing the unique needs of frail individuals, potentially reducing mortality rates and preventing unnecessary readmissions. Ultimately, this could enhance the quality of care for older adults and alleviate the strain on emergency healthcare resources. Moreover, our results suggest that frailty assessment may be a valuable addition to the toolkit of emergency physicians, enabling more informed decision-making regarding patient disposition and care plans. We actively seek to disseminate our findings and collaborate with healthcare stakeholders to translate this knowledge into tangible improvements in practice and enhance the well-being of older patients seeking emergency care.

In the literature, other frailty scales such as the ISAR score [12,13,14,15,16,17,18,19], TRST [20], and CFS [21] have been evaluated for use in the emergency department. Our study adds to this discussion by demonstrating the potential of the mSEGA frailty scale in identifying patients at risk for adverse outcomes within this specific setting.

In our study, we aimed to explore the predictive capacity of the mSEGA frailty scale for morbidity and mortality. To provide a comparative perspective, we also utilized the Charlson Comorbidity Index to assess the association between the evolving concept of frailty and the medical reality of comorbidities within the same sample.

Our findings revealed that the Charlson Comorbidity Index exhibited a significant capacity to predict mortality at 12 months (*p* < 0.0001). However, it did not demonstrate predictive abilities for readmission to the emergency department, institutionalization, or initiation of home care. On the other hand, the mSEGA frailty scale, represented by the SEGA grid, exhibited a significant capacity to predict readmission to the emergency department at 12 months (*p* = 0.005). However, it did not demonstrate significant predictive capacity for mortality, institutionalization, or initiation of home care.

It is interesting to note that the health status of patients considered through their comorbidities via the Charlson Comorbidity Index is not superimposable on the degree of frailty according to the mSEGA frailty scale. It would be easy to conclude that patients with multiple comorbidities are necessarily frail and are, therefore, at risk of death, but this is not the case; our study shows that there is no obvious correlation between patients considered frail or very frail according to the mSEGA frailty scale, and a high Charlson comorbidity score.

Our pilot study showed that the mSEGA frailty scale provides prognostic information regarding readmission to the emergency department within one year of the initial admission, whereas the Charlson Comorbidity Index provides prognostic information regarding the risk of death at one year. Because of its multidimensional character (comorbidities, physical, cognitive, mood changes), the mSEGA frailty scale may replace the comprehensive gerontological assessment conducted in the emergency department and provide specific, systematized elements to an associated geriatric team, particularly when working together with a mobile geriatric team, making it possible to anticipate the adverse events that lead to patients being readmitted to the emergency department.

### Limitations

The study has very important limitations, derived from the opportunistic selection of cases (only 255 patients in 7 months suggests a large selection bias), and the retrospective nature of the study. This study’s limitations include its retrospective nature, reliance on medical records for data collection, and potential incomplete information due to issues like professional confidentiality and lack of communication between hospitals. Additionally, the study’s use of older data may limit its applicability to current healthcare practices. Furthermore, the study’s relatively short follow-up duration may have affected the results by not capturing longer-term outcomes. The opportunistic selection of cases led to an important bias in the selection of cases. It may have limitations in terms of sample size compared to more structured recruitment methods.

## 5. Conclusions

In conclusion, our pilot study underscores the potential significance of frailty assessment as a predictive tool in the emergency department. Although we lack specific references to support our findings, the statistically significant results related to mortality and readmission outcomes highlight the potential importance of frailty assessment in improving patient care for older adults. Further research and validation studies are warranted to confirm these preliminary findings and establish a more solid evidence base.

## Figures and Tables

**Table 1:** Description of the population at admission (*n* = 255) included from 1 July 2017 to 31 January 2018.

Variables	Total N = 255
Age, mean ± SD, years	82.1 ± 8.2
Sex	
Female	145/255 (56.9)
Male	110/255 (43.1)
Method of referral to emergency department	
Patient	61/254 (24.0)
Attending physician	54/254 (21.3)
Emergency call center	134/254 (52.8)
Other	5/254 (2.0)
Chaumont Hospital Emergency Dept.	1/5 (20.0)
Nursing home	1/5 (20.0)
Doctor on call	2/5 (40.0)
Out-patient care nurse	1/5 (20.0)
Reason for admission to the emergency room	
Fall	51/254 (20.0)
Altered general condition	28/255 (11.0)
Digestive (excluding digestive bleeding)	30/255 (11.8)
Digestive bleeding	3/255 (1.2)
Chest pain	16/255 (6.3)
Dyspnea	26/255 (10.2)
Malaise	18/255 (7.1)
Neurological	14/255 (5.5)
External bleeding	8/255 (3.1)
Other	61/255 (23.9)
mSEGA ^1^ frailty scale	
Low-frailty patients	78/255 (30.6)
Frail patients	49/255 (19.2)
Very frail patients	128/255 (50.2)
Charlson score, mean ± SD	5.5 ± 2.0
Medication, median (EIQ)	8 (5–10)
Polypharmacy ^2^	182/253 (71.9)
Institutional living	44/255 (17.2)
Home care	90/255 (35.3)
Orientation after admission	
Returned home	103/255 (40.4)
Hospitalization	152/255 (59.6)

The data show the number (%) otherwise indicated. IQR = interquartile range; SD = standard deviation. mSEGA = modified Summary Geriatric Profile Evaluation at Admission. ^1^ A SEGA score ≤8 defines low-frailty; a score between 9 and 11 defines frail; a score >11 defines very frail. ^2^ Defined by a number of treatments ≥ 6.

**Table 2:** Evaluation criteria.

	Total N = 255	Frail/Very Frail Patients N = 177	Low-Frailty Patients N = 78	*p*-Value
Institutionalization within 12 months ^1^	11/210 (5.2)	10/132 (7.6)	1/78 (1.3)	0.06
Home care initiated within 12 months ^2^	19/120 (15.8)	9/48 (18.7)	10/72 (13.9)	0.47
Deaths ^3^	63/255 (24.7)	53/177 (29.9)	10/78 (12.8)	0.004
Emergency readmission	30/62 (48.4)	26/52 (50.0)	4/10 (40.0)	0.61
Without emergency readmission	32/62 (51.6)	26/52 (50.0)	6/10 (60.0)	
Possible readmission to emergency department during 12 months	71/254 (27.9)	59/176 (33.5)	12/78 (15.4)	0.003

^1^ Among the population not living in an institution at admission and still living there one year later; ^2^ the population without home care at admission and who were still receiving home care one year later; ^3^ the *p*-value corresponds to a log-rank test; The data are presented as a number (%).

**Table 3:** Univariate analysis of factors associated with institutionalization within 12 months of the visit to the emergency department.

	Institutionalization N = 11	Not Institutionalized N = 199	*p*-Value
Frailty			0.06
Low-frailty patients	1/11 (9.1)	77/199 (38.7)	
Frail/very frail patients	10/11 (90.9)	122/199 (61.3)	
Age, mean ± SD, years	83.3 ± 6.5	80.5 ± 7.9	0.25
Sex			0.84
Female	6/11 (54.5)	102/198 (51.5)	
Male	5/11 (45.5)	96/198 (48.5)	
Charlson score, mean ± SD	5.5 ± 1.5	5.3 ± 2.1	0.84
Polypharmacy	8/11 (72.7)	138/197 (94.7)	1.00

The data show the number (%). SD = standard deviation.

**Table 4:** Univariate analysis of factors associated with initiation of home care within 12 months of emergency room visit.

	Home Care Provided N = 19	Home Care Not Provided N = 101	*p*-Value
Frailty			0.47
Low-frailty patients	10/19 (52.6)	62/101 (61.4)	
Frail/very frail patients	9/19 (47.4)	39/101 (38.6)	
Age, mean ± SD, years	80.6 ± 6.4	78.0 ± 7.7	0.17
Sex			0.98
Female	9/19 (47.4)	47/100 (47.0)	
Male	10/19 (52.6)	53/100 (53.0)	
Charlson score, mean ± SD	4.4 ± 1.0	4.7 ± 1.7	0.52
Polypharmacy	11/19 (61.1)	62/101 (61.4)	0.98

The data show the number (%). SD = standard deviation.

**Table 5:** Univariate and multivariate analysis of factors associated with readmission during the 12 months after the emergency room visit.

	Readmission N = 71	Not Readmitted N = 183	Univariate Analysis	Multivariate Analysis	
			*p*-Value	Adjusted OR ^1^(CI 95%)	*p*-Value
Frailty			0.003		0.005
Low-frailty patients	12 (16.9)	66 (36.1)		1.00 (ref)	
Frail/very frail patients	59 (83.1)	117 (63.9)		2.71 (1.36–5.41)	
Age, mean ± SD, years	82.4 ± 8.5	81.9 ± 8.1	0.69	-	-
Sex			0.81		-
Female	39 (55.7)	105 (57.4)		-	
Male	31 (44.3)	78 (42.6)		-	
Charlson score, mean ± SD	5.9 ± 2.4	5.4 ± 1.8	0.07	-	-
Polypharmacy	56 (80.0)	125 (68.7)	0.07	-	-

The data show the number (%). SD = standard deviation; OR = odds-ratio; CI = confidence interval. ^1^ The variables included in the multivariate top-down logistic regression model were frailty, Charlson score, and polypharmacy.

**Table 6:** Univariate and multivariate analysis of factors associated with death during the 12 months after emergency room visit.

	Deaths	Univariate Analysis		Multivariate Analysis	
	Number (%) ^1^	HR (CI 95%)	*p*-Value	Adjusted HR ^2^ (CI 95%)	*p*-Value
Frailty			0.006		-
Low-frailty patients	10 (12.8)	1.00 (ref)		-	
Frail/very frail patients	53 (29.9)	2.60 (1.32–5.10)		-	
Age	-	1.04 (1.01–1.07)	0.02	-	-
Sex			0.33		-
Female	31 (28.4)	1.00 (ref)		-	
Male	32 (22.1)	0.78 (0.48–1.28)		-	
Charlson score	-	1.25 (1.13–1.39)	<0.0001	1.26 (1.14–1.39)	<0.0001
Polypharmacy			0.03		-
No	11 (15.5)	1.00 (ref)		-	
Yes	52 (28.6)	2.02 (1.06–3.88)		-	

CI = confidence interval. ^1^ The data is the number (Kaplan–Meier estimation). ^2^ Variables included in the Cox multivariate proportional hazards model with top-down variable selection are frailty, age, Charlson score. and polypharmacy.

## Data Availability

The datasets used and/or analyzed during the current study are available from the corresponding author upon reasonable request.
